# Community case management in malaria: review and perspectives after four years of operational experience in Saraya district, south-east Senegal

**DOI:** 10.1186/1475-2875-12-240

**Published:** 2013-07-12

**Authors:** Youssoupha Ndiaye, Jean LA Ndiaye, Badara Cisse, Demetri Blanas, Jonas Bassene, Isaac A Manga, Mansour Ndiath, Sylvain L Faye, Mamoudou Bocoum, Mouhamed Ndiaye, Pape M Thior, Doudou Sene, Paul Milligan, Omar Gaye, David Schellenberg

**Affiliations:** 1 Ministry of Health and Social Action, Dakar, Senegal; 2 Department of Medical Parasitology, University Cheikh Anta Diop, Dakar, Senegal; 3 Department of Sociology, University Cheikh Anta Diop, Dakar, Senegal; 4 Department of Family Medicine, Icahn School of Medicine at Mount Sinai, New York, USA; 5 Health Service Division, Oromin Joint Venture Group, Sabodala, Senegal; 6 Department of Infectious Disease Epidemiology, London School of Hygiene and Tropical Medicine, London, UK; 7 Department of Disease Control, London School of Hygiene and Tropical Medicine, London, UK

**Keywords:** Malaria, Community case management, Community health

## Abstract

**Background:**

Despite recent advances in malaria diagnosis and treatment, many isolated communities in rural settings continue to lack access to these life-saving tools. Community-case management of malaria (CCMm), consisting of lay health workers (LHWs) using malaria rapid diagnostic tests (RDTs) and artemisinin-based combination therapy (ACT) in their villages, can address this disparity.

**Methods:**

This study examined routine reporting data from a CCMm programme between 2008 and 2011 in Saraya, a rural district in Senegal, and assessed its impact on timely access to rapid diagnostic tests and ACT.

**Results:**

There was a seven-fold increase in the number of LHWs providing care and in the number of patients seen. LHW engagement in the CCM programme varied seasonally, 24,3% of all patients prescribed an ACT had a negative RDT or were never administered an RDT, and less than half of patients with absolute indications for referral (severe symptoms, age under two months and pregnancy) were referred. There were few stock-outs.

**Discussion:**

This CCMm programme successfully increased the number of patients with access to RDT and ACT, but further investigation is required to identify the cause for over-prescription, and low rates of referrals for patients with absolute indications. In contrast, previous widespread stock-outs in Saraya’s CCMm programme have now been resolved.

**Conclusion:**

This study demonstrates the potential for CCMm programmes to substantially increase access to life-saving malarial diagnostics and treatment, but also highlights important challenges in ensuring quality.

## Background

Indicators of malaria morbidity and child survival have significantly improved in most African countries, following the widespread implementation of control strategies [1], including measures to increase the use of insecticide-treated bed nets, the introduction of artemisinin-based combination therapy (ACT) and in some areas, the use of rapid diagnostic tests (RDTs) for malaria, allowing effective treatment to be targeted to the most needy. However, access to these new diagnostic and treatment tools is primarily limited to health posts and health centres. Extending appropriate and effective care to remote and sparsely populated areas is a major challenge due to a shortage of formally trained health professionals [2,3], among other reasons. Lack of access to care also limits the availability of data on the magnitude of the disease burden in these areas. To overcome these barriers, diverse strategies have been developed, most notably those expressed in the Declaration of Alma Ata, which endorses community health workers (CHWs) and traditional healers as important health care providers in resource-limited settings [4].

The Senegalese Ministry of Health began integrating CHWs into the health care system in 1977 with the Sine Saloum rural project [5]. This project’s objective was to establish 200 health huts, run by village volunteers trained to provide basic health care, including routine immunizations, raising community awareness for malaria, oral rehydration therapy and childhood development monitoring [5]. Since then, CHWs have been involved in several other programmes including immunization campaigns, the integrated management of childhood illness or reproductive health programmes [6,7].

In 2007, the National Malaria Control Programme (NMCP) integrated ACT and RDTs into the community case management of malaria (CCMm) programme, called PECADOM (*Prise en charge des cas de paludisme à domicile)*, and implemented it nationwide from 2008 [8,9]. The Ministry of Health authorized CHWs to use RDTs and prescribe anti-malarial drugs in villages that have health huts, which are small health facilities typically with one or two rooms and often staffed with a community birth attendant. The CHWs offer malaria diagnosis and treatment, diarrhoea treatment, pneumonia case management with an antibiotic, and other services which may include growth monitoring and promotion, deworming, vitamin A supplementation, malnutrition management, family planning and reproductive health services. In certain villages without a health hut, volunteers are trained to use only RDTs and prescribe ACT to patients with uncomplicated cases of malaria. These malaria volunteers, known locally as *Distributeurs de Soins à Domicile* (DSDOM) will be referred to in this paper as Community Medicine Distributors (CMDs). CHWs and CMDs will be referred to collectively as lay health workers (LHWs). These strategies were developed in an attempt to increase access to care in under-served areas [10]. This paper reviews the development of the CCMm in Saraya, rural Senegal, presents findings from an evaluation of the programme and defines future research and programme priorities.

## Methods

### Study area

**Figure 1:**
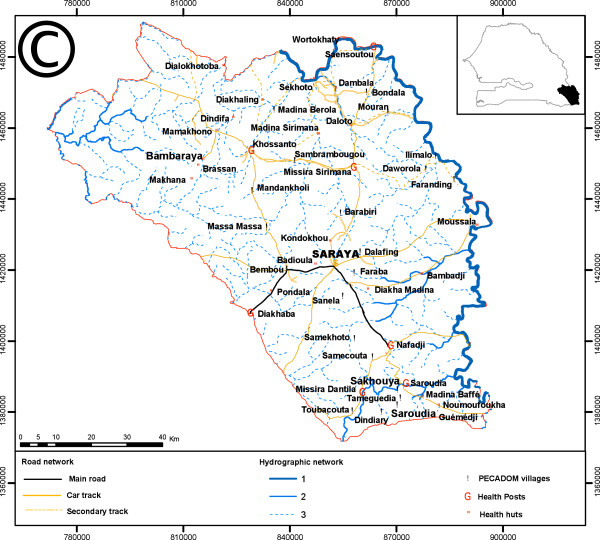
Saraya in Senegal map.

Malaria is highly endemic and seasonal in Saraya, a district in south-eastern Senegal nearly 800 km from the capital, Dakar. Saraya borders both Guinea and Mali (Figure 1). It has a population density of seven inhabitants per sq km distributed over 6,800 sq km with an estimated population of 40,000 persons. This population estimate is based on the most recent national census updated to allow for population growth, but does not reflect recent increases in regional immigration due to the rapid growth of traditional gold mining in the area. The ethnic groups in Saraya consist primarily of Malinke, Fulas, Diallonkes and Diakhanke. The road network is sparse and rivers limit access to many villages in the rainy season. In 2008, the district health service consisted of the district health centre, seven health posts and five health huts. Over 70% of the population lives more than five km from a health post or health centre.

Mortality among children under five years of age in the region of Kedougou (in which Saraya is a district) is estimated at 154 deaths per 1,000 live births [11]. This is the highest mortality rate in Senegal. The average national rate in 2010-2011from a demographic and health survey and multiple indicators cluster survey estimated under five mortality rate to be at 72 deaths per 1,000 live births. The Kedougou region also has the highest prevalence of *Plasmodium falciparum* among children less than five years of age of 14%, compared to the national average of 3%. The rate of stunting due to malnutrition is also high at 39% compared to the national average of 27% [11].

### Design of the community case management programme

The district management team used a community consensus process involving community leaders to select CHWs with selection criteria based on French reading and writing ability, permanent residence in the village, and willingness to support the community on a voluntary basis.

Although not being official health personnel, CHWs are trained by the district offices to carry out specific tasks within approved health programmes, such as the Expanded Programme on Immunization, the management of respiratory infections, malaria, reproductive health and HIV, tuberculosis, nutrition, water and sanitation, education, communication, and social mobilization. CHWs only provide treatment in uncomplicated malaria, respiratory infections and diarrhoea and must promptly refer patients beyond their level of training to the nearest health as per Ministry of Health protocol. The classroom component of their CCMm training lasted approximately one month, and was hosted and supervised by the district health office. The CHWs were taught to diagnose and treat specific uncomplicated conditions using a Ministry of Health algorithm that directs them when it is appropriate to refer. Training on reporting monthly activities, referring complicated conditions and monitoring pharmacovigilance was also provided.

After their stay in the health centre, CHWs were assigned for field training to the health post covering their village for up to a month. At the health post, the CHWs improved their skills and knowledge in malaria, diarrhoea and acute respiratory infection diagnostis, treatment and prompt referral under nurses’ supervision.

Following the introduction of RDTs at the community level in 2008, CMDs were chosen through the same process of community consensus. Nurses trained them to recognize the symptoms of malaria, to use RDTs and ACT, and to recognize drug-related adverse events. After three days of classroom teaching at the district health centre, they received an additional 15 days of training in the health post in their catchment area. The training material was based on the NMCP model, with subjective fever as the main indication to performing an RDT as the programme did not provide thermometers to community personnel. The national and regional pharmacy provided ACT free of charge when requested by the government district health service. CHWs and CMDs alerted the district management team chief nurse when they required supplies, and were provided with them either when they visited the district health centre or when the district management team vehicle visited their village. The CHWs stored RDTs, ACT and other drugs in secure locations in health huts. The NMCP provided suitcases and wooden boxes to the CMDs to store the ACT and RDTs.

CHWs and CMDs were trained to provide monthly reports with summaries of patient information, including name, age, gender, address, symptoms, RDT result if an RDT was used, diagnosis, treatment (e g, ACT use), any suspected adverse drug reactions, referrals, and deaths in their village. Additional case notification report forms were distributed in 2010 to be used for more detailed recording of deaths and suspected adverse drug reactions, including abdominal pain, vomiting, cutaneous rash and toxic epidermal necrosis. The adverse event monitoring form was adapted from an NMCP model. Mortality surveillance was carried out through information collected by CHWs and CMDs in the community. Nurses and experienced CHWs and CMDs piloted and validated the forms at a workshop. The LHWs were required to send the adverse event forms to the local nurse who evaluated the cases and sent official notification to the Ministry of Health via the district management team. In 2010 and 2011, refresher training was provided to the LHWs.

### Referral system

CHW training emphasized that patients less than two months of age, patients with suspected medication adverse events, patients with negative RDT, and patients with severe cases of malaria, diarrhoea or acute respiratory illness, should be referred to the nearest health post. CMDs were trained to refer all cases except for those with RDT-confirmed uncomplicated malaria. The research team provided the LHWs with a referral form to be presented by the patient to the health post staff. The receiving provider completed a duplicate referral form, which was given to the patient in order to provide feedback to the CHW or CMD.

### Quality assurance

The research team assessed CHW and CMD performance using a quality assurance system, based on the triangulation of sources of information namely: (i) direct observation and home visits to patients or caregivers, and (ii) supervision by the DMT.

### Direct observations and home visits

Throughout 2010 and 2011, supervisors observed the LHWs in their health hut or rooms as they carried out each step of the patient encounter, from welcoming patients to providing them with recommendations. The observations included cleanliness of the sampling area on the fingertip, proper technique in performing the diagnostic test, appropriate ACT administration according to the Ministry of Health flowchart, proper patient counselling on the signs of severe malaria and other methods of malaria prevention. The observers conducted same-day home visits for the last three patients seen by the CHW or CMD as identified from the lay health worker register. During these home visits, the observer questioned patients or caregivers on the same topics. These observation visits were most often conducted at different time as the LHW supervisions described below.

### CHW and CMD routine supervision

The supervision protocol was adapted from the NMCP model and ensured several levels of supervision, including community supervisors, nurses and district health team staff. The objective of the supervision visit was to monitor the drug supply system, medication-associated adverse events, deaths, referrals and community involvement. The research team developed an assessment system to evaluate the availability of ACT, acetaminophen, RDTs, gloves, thermometer and stock spread sheet as well as appropriateness of treatment and referrals with correct treatment defined as the proper ACT dose administered after a positive RDT. The research team assessed if the LHWs correctly made referrals based on the RDT protocol algorithm. The supervisor discussed the management of adverse events and deaths with the LHWs and assessed RDT technique in 2010. The supervisor marked each step as “satisfactory”, “unsatisfactory” or “not applicable”. Finally, the supervisor evaluated community involvement by interviewing the village chief. If the community leaders were involved in the CCMm programme, especially in the selection of LHWs, this was marked as 1 and as 0 if they were not.

### Ethics

The study protocol was reviewed and approved by the National Committee for Ethics and Research in Senegal. Informed consent was obtained from community leaders, parents and caregivers.

### Data analysis

Data were entered in Excel and analysed using Stata 11® and Excel® 2007. The data was independently analysed in SPSS to cross-check for accuracy.

## Results

### Increased access and seasonal variation in CCMm service availability

**Figure 2:**
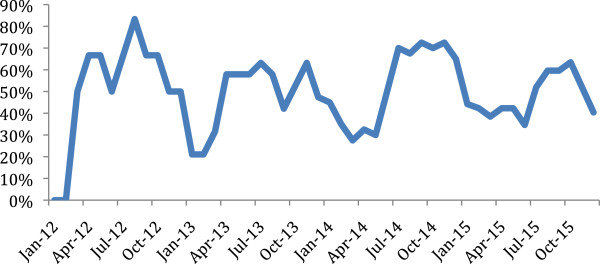
**Proportion of trained LHWs actively working in their village over time.** Legend: More LHWs engaged in CCMm activities during the rainy season.

The number of LHWs trained in the CCMm programme increased from six to 52 between 2008 and 2011 (Table 1). In 2008, 1,298 consultations were completed by six LHWs covering and estimated population of 3,471; by 2011, this had increased to 9,617 visits completed by 43 LHWs covering an estimated population of 19,505. This showed a six-fold increase in the number of actively working LHWs and LHW patient visits. However, LHW involvement in the CCMm programme varied seasonally with more trained LHWs actively providing services during the rainy season from July to November than during other periods (Figure 2).

**Table 1:** Population served by a CHW or CMD

	**2008**	**2009**	**2010**	**2011**
# of CHWs trained	6	11	20	21
# of CMDs trained	0	8	21	31
Total # of LHWs trained	6	19	41	52
# of villages where LHW is actively engaged in CCMm programme activities	6	19	40	43
Annual patient encounters	1,298	2,514	8,732	9,597
Population in villages with a CCMm-trained LHW by year	3,471	6,915	17,360	19,505

### CHW and CMD clinical activities

#### Patient demographics

LHWs saw a total of 22,141 patients between 2008 and 2011 (Table 2). Women made up approximately half (47.5%, n = 10,512), and children less than five years of age more than a third (36.9%, n = 8,171) of all patients. CHWs saw approximately three times more patients than CMDs (16,757 vs. 5,384 patients).

**Table 2:** Lay health worker activity

	**CHW**	**CMD**	**Total**
Total consultations (% of all consultations)	16,757 (75.7%)	5,384 (24.3%)	22,141 (100%)
Women patients (%)	7,994 (47.7%)	2,527 (46.9%)	10,521 (47.5%)
Children <5 yrs of age (%)	6,201 (37.0%)	1,970 (36.6%)	8,171 (36.9%)
Total RDTs conducted (% of all consultations)	9,518 (56.8%)	5,036 (93.5%)	14,554 (65.7%)
RDT + ve (%)	6,800 (71.4%)	3,800 (75.5%)	10,600 (72.8%)
RDT –ve (%)	2,718 (28.6%)	1,236 (24.5%)	3,954 (27.2%)
Total ACT prescribed (% all consultations)	8,756 (52.3%)	3,779 (70.2%)	10,600 (47.9%)
No. RDT + ve prescribed an ACT (% RDT+)	6,129 (70.0%)	3621 (95.3%)	9750 (84.9%)
No. RDT –ve prescribed an ACT (% RDT-)	675 (7.7%)	128 (3.4%)	803 (7.0%)
No. prescribed ACT without RDT (% all ACTs)	1,952 (22.3%)	30 (0.8%)	1982 (17.3%)
Total referrals (% of all consultations)	1,860 (11.1%)	1187 (22.0)	3,047 (13.8%)
RDT –ve (%)	806 (43.3%)	1047 (88.2%)	1,853 (60.8%)
RDT + ve (%)	137 (7.4%)	57 (4.8%)	194 (6.4%)
No RDT performed (%)	919 (49.4%)	83 (7.0%)	1002 (32.9%)
Severe symptoms (%)	112 (6.0%)	5 (0.4%)	117 (3.8%)
Child under 2 months (%)	33 (1.8%)	12 (1%)	45 (1.5.0%)
Pregnant woman (%)	7 (0.4%)	1 (0.1%)	8 (0.3%)
Other reason for referral (%)	105 (5.6%)	63 (5.3%)	168 (5.5%)

#### RDT and ACT use

In total, LHWs confirmed 10,600 cases of malaria by RDT, and prescribed 11,488 treatments with ACT. CHWs performed proportionally fewer RDTs than CMDs (56.8% vs. 93.5%); however, the percentage of RDTs that were positive was similar in both groups (71.4% vs. 75.5% respectively). CHWs also provided ACT to a smaller percentage of patients than CMDs (53.2% vs. 70.2%), but prescribed ACT to a larger percentage of patients with negative RDTs (24.8% vs. 10.4 %) and to patients who had never received an RDT (22.3% vs. 0.8%) than did CMDs.

#### Patient age distribution

**Figure 3:**
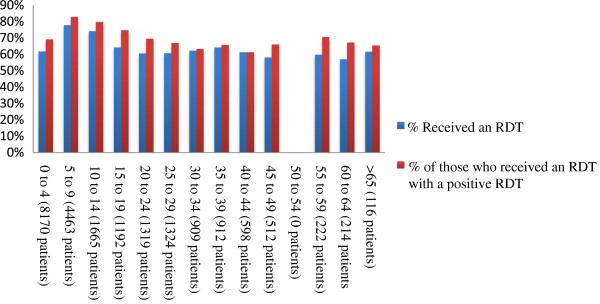
**Proportion of patient receiving an RDT and proportion with a positive RDT by age.** Legend: The overall number and proportion of patients receiving an RDT and the proportion with a positive RDT were higher among younger age groups.

Over half (61.0 %, n = 6,376) of patients with a positive RDT were children under 10 years of age and more than a third (33.4%, n = 3,495) were under five years of age. The proportions of patients administered an RDT and with a positive RDT were also higher among younger age groups (Figure 3).

#### Referrals

The LHWs documented 3,047 referrals in the registers, representing 13.8% of all consultations. The most common reason for referrals was a negative RDT (60.8%, n = 1,853), and most of these (56.5%, n = 1,047) were referrals made by CMDs. Overall CHWs referred proportionally fewer patients than did CMDs (11.1% vs. 22.0%), and fewer patients with a negative RDT (43.3% vs. 88.2%), but referred proportionally more patients who had not received an RDT (49,4% vs. 7.0%). The difference in the proportion of patients referred by CHWs or CMDs for severe symptoms (6.0% vs. 0.4%), for age less than two months (1.8% vs. 1.0%), or pregnancy (0.4% vs. 0.1%) was small. However, less than half of all patients with severe symptoms (25.9%, n = 117), less than a third of all patients with severe symptoms (25.9%, n = 117), less than a third of all patients two months of age or younger (18.2%, n = 45), and less than half of all pregnant patients (47.1%, n = 8) were referred by either the CHWs or the CMDs.

### Comparison of CCMm villages and Government health posts

**Figure 4:**
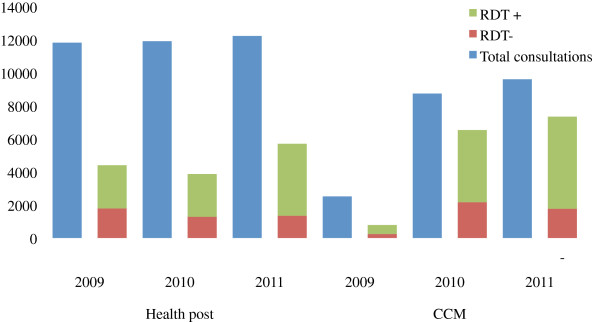
Health Post vs. CCM Patient and RDT Volume.

According to routine monthly reports, in 2009, district health nurses saw approximately five times more patients than LHWs (11,812 vs. 2514), and performed more RDT than LHWs (4,402 vs. 786) (Figure 4). However, by 2011, although district nurses still saw more patients than LHWs (12,216 vs. 9,596), nurses performed a quarter fewer RDT than LHWs (5,698 vs. 7,337). The proportion of positive RDT was approximately the same between the two groups and over time, and the number of patients seen or RDTs performed by nurses did not decrease as LHW patient volume increased.

### CHW and CMD surveillance of adverse events and deaths

One adverse event (vomiting and abdominal pain) following anti-malarial medication was reported. Overall, 14 LHWs reported 20 deaths, of which 12 were under five years of age, five were between five and 10 years, and three were older than 10 years.

### Quality assurance

#### Direct observations and home visits

**Figure 5:**
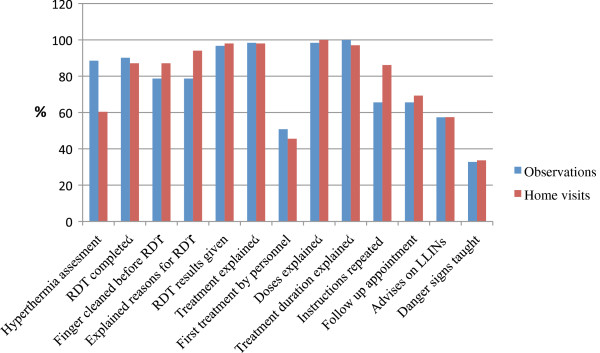
Responses rate to items monitored during observation and home visits sessions.

In 2010 and 2011, district management team supervisors and two members of the research team performed 61 observations with CHWs (48 observations) and CMDs (13 observations), and 101 home visits of patients seen by CHWs (60 patients) and CMDs (41 patients) (Figure 5). Fifty-four of the 61 LHWs recorded subjective fever before administering an RDT. Nearly all LHW told their patients the adequate dosage and duration of treatment prior to receiving treatment. Only 31 LHWs directly observed the patient take the first ACT dose in their health facility. Sixty of the 101 patients visited at home reported having had a subjective fever prior to being administered the RDT. Follow-up appointments and counselling on prevention and recognition of danger signs were rarely carried out.

### CHW and CMD supervision

Supervisors completed 51 visits among CHWs (28) and CMDs (23) during the rainy seasons of 2010 and 2011. They noted few stock-outs with 46 of the 51 LHWs (90.2%) having RDTs in stock, 45 (88%) having sufficient ACT, and 35 (68.6%) having adequate supplies of paracetamol. Ancillary supplies including gloves (available at 22 [43%] sites), thermometers (23 [45.1%] sites), and stock tracking forms (11 [21.6%] sites, of which only 6 [11.8%] forms were completed), were less available. All CHWs and CMDs knew the correct ACT treatment dose duration. They informed supervisors of 38 deaths in the two months prior to the supervision occurring either in the village, health post, or health centre. LHWs attributed 16 of these fatalities to malaria based on a positive RDT prior to death, and information from health staff; however, LHWs had notified nurses of only 11 of these deaths. CHWs and CMDs performed RDTs with an acceptable skill level (Table 3), but hygiene standard were frequently not met, in part as a result of a 2010 shortage in gloves due to delays in the procurement system.

**Table 3:** Quality of RDT performance by CHWs and CMDs

	**Good practices n (%)**	**Practices to improve (%)**
Surface is clean and flat	26 (87)	4 (13)
Tests opened just before use	30 (100)	0 (0)
Documented patient name and date	25 (83)	5 (17)
Use of gloves	0 (0)	30 (100)
5 microliters (5 μL) finger prick blood specimen	28 (93)	2 (7)
Deposited 5 μL of blood in well A	28 (93)	2 (7)
Deposited 4 drops of solution vertically in well B	28 (93)	2 (7)
Let the test rest on a level surface	29 (97)	1 (3)
Waited a maximum of 15 minutes for the result	28 (93)	2 (7)

## Discussion

The feasibility and acceptability of LHWs administering RDTs and ACT in non-severe malaria within CCMm programmes has been demonstrated previously [5,12-16]. This study assessed LHWs performance in a rural malaria-endemic district between 2008 and 2011 and sought to identify priorities for future improvement in policy and implementation. The major findings include (i) a large increase in the number of trained LHWs offering CCMm services and patients benefitting from these services, but seasonal variations in the number of actively working LHWs, (ii) an excess of ACT courses prescribed over positive RDTs particularly among CHWs, (iii) a majority of referrals were made after a negative RDT, but few patients with absolute indications for referral (severe symptoms, ≤ 2 months of age or pregnancy) were referred, and (iv) there were few stock outs. A discussion of these findings and their implications for future work is presented below.

### Increased access to RDT and ACT services

A seven-fold increase was observed in the number of LHWs trained and the number of patients visited from 2008 to 2011. It does not appear that this increase relieved district health posts nurses of excess patient burden as has been documented in other studies [10], as the nurses in Saraya continued to see the same number of patients over the course of the study. It does suggest, however that the greater number of LHWs increased overall access to RDTs and ACT treatment as reflected in the higher number of patients diagnosed and treated. This result was achieved in part through the advocacy and commitment of the district health team, community leaders, local authorities and a mining company that built one third of the health huts in the area, and highlights the importance of local engagement in the success of nationally implemented community programmes.

An additional important finding was seasonal variation in engagement of LHWs in the CCMm programme in their village with greater engagement during the rainy season. Possible explanations include the higher burden of malaria and the attraction of work in nearby traditional gold mining site, in combination with the lack of LHWs remuneration for CCMm programme activities. These conflicting interests faced by the LHWs has been documented elsewhere [17], and further qualitative investigation should be conducted into their causes and potential solutions in Saraya.

### Excess of ACT courses prescribed over RDTs administered

The comparison of RDT results with ACT administration revealed that almost a third of patients who received ACT had a negative RDT or did not receive an RDT at all. However these rates varied between CHWs and CMDs with CHWs prescribing ACT to a quarter of all patients with a negative RDT, compared to CMDs who prescribed ACT to only one out of every ten patients with a negative RDT. The reasons for this deviation from the CCMm algorithm may be due to LHWs scepticism of RDT sensitivity, or RDT stock-outs and resulting presumptive treatment. The reason for the discrepancy between rates of excess treatment between CHWs and CMDs is also not known, and it contrasts with other publications, which report low rates of ACT administration to patients with negative RDTs [18,19]. The potential consequences of this overtreatment include patient dissatisfaction, accelerated emergence of ACT resistance, and increased cost [20], and thus warrants future investigation.

### Majority of referrals were appropriate, but few high-risk patients were referred

The majority of patients who were referred had a negative RDT, but CMDs referred proportionally more patients with a negative RDT than did CHWs. This difference may be due to the fact that CHWs are trained to treat other conditions, whereas CMDs are trained to refer all patients with a negative RDT. Restricting CMD responsibilities to malaria may increase the risk of over-diagnosis and treatment, and the progression to severe acute febrile illness among children less than 10 years, who represent more than 50% of patients. Previous studies have discussed the risk of misdiagnosis due to overlapping symptoms between pneumonia and malaria in programmes for malaria treatment alone [21,22]. Careful consideration should be given to increasing the scope of CMD responsibilities to include uncomplicated pneumonia similarly to CHWs [23] while also taking into account the cost of training and quality control.

Another concerning finding in this study was that only a minority of patients with absolute indications for referral (including severe symptoms, age under two months, and pregnancy) were referred. This is in contrast to other studies of CCMm programmes that found high rates of appropriate referral for patients with severe malaria [18]. The low rates of referral of high risks patients in Saraya may be due to financial and logistical barriers, especially during the rainy season and warrants further investigation.

### Few stock-outs

In contrast to a previous assessment of the CCMm programme in Saraya which found wide-spread stock-outs of ACT and RDTs soon after the programme’s implementation [24], this study documented overall availability of these supplies suggesting that early logistical challenges have already been overcome.

## Conclusion

This study examines routine reporting data of a CCMm programme in rural south-east Senegal. It documents a large increase in the number of patients with access to RDT and ACT; however, it also identifies aspects of the CCMm programme that can be improved including over-prescription of ACTs to patients with a negative RDT or who had not received an RDT at all, and low referral rates of patients with absolute indications for referral including severe symptoms, age under two months, or pregnancy. In contrast, the analysis demonstrated that ACT and RDT stock-outs, which had previously been identified as a widespread problem, had largely been eradicated. Although this study demonstrates the need for on-going efforts to improve LHW performance and to investigate referral barriers in Saraya’s CCMm programme, the LHWs’ success in increasing access to life saving care testifies lay health workers’ important role in community health programmes as first described in the Declaration of Alma Ata.

## Consent

Written informed consent was obtained from the patient’s guardian / parent / next of kin for the publication of this report and any accompanying images.

## Competing interests

The authors declare that they have no competing interests.

## Authors’ contributions

YN coordinated the study. YN, BC, JLN, OG and DSc designed the study. YN and BC wrote the protocol. DS, PMT and JLN revised protocol. JB, AM, MN, SF and MB assisted in implementation, monitoring and data collection YN, DB and PM analysed data. YN wrote the manuscript. DSc, BC, PM, DB and JLN revised the manuscript. All authors read and approved the final manuscript.
